# Frequency and risk factors for naturally occurring Cushing's syndrome in dogs attending UK primary‐care practices

**DOI:** 10.1111/jsap.13450

**Published:** 2021-12-08

**Authors:** I. Schofield, D. C. Brodbelt, S. J. M. Niessen, D. B. Church, R. F. Geddes, D. G. O'Neill

**Affiliations:** ^1^ Pathobiology and Population Sciences The Royal Veterinary College Hatfield, Herts AL9 7TA UK; ^2^ Clinical Science and Services The Royal Veterinary College Hatfield, Herts AL9 7TA UK; ^3^ Veterinary Specialist Consultations Hilversum The Netherlands

## Abstract

**Objectives:**

To estimate the frequency and risk factors for Cushing's syndrome in dogs under UK primary veterinary care.

**Materials and Methods:**

Dogs with Cushing's syndrome were identified by searching electronic patient records of primary‐care veterinary practices. Pre‐existing and incident cases of Cushing's syndrome during 2016 were included to estimate the 1‐year period prevalence. Incident cases were used to estimate the annual incidence and to identify demographic risk factors for the diagnosis of Cushing's syndrome in dogs, through multivariable logistic regression.

**Results:**

Analysis included 970 pre‐existing and 557 incident cases of Cushing's syndrome from a population of 905,544 dogs. The estimated 1‐year period prevalence for Cushing's syndrome in dogs under veterinary care was 0.17% (95% confidence interval 0.16 to 0.18) and incidence was 0.06% (95% confidence interval 0.05 to 0.07). In multivariable logistic regression modelling, the Bichon frise (odds ratio=6.17, 95% confidence interval 4.22 to 9.00), Border terrier (5.40, 95% confidence interval 3.66 to 7.97) and Miniature schnauzer (3.05, 95% confidence interval 1.67 to 5.57) had the highest odds of Cushing's syndrome. The Golden retriever (0.24, 95% confidence interval 0.06 to 0.98) and Labrador retriever (0.30, 95% confidence interval 0.17 to 0.54) were the most protected breeds. Increasing age, bodyweight greater than the breed‐sex mean and being insured also showed increased odds of Cushing's syndrome.

**Clinical Significance:**

As Cushing's syndrome is predominately diagnosed and managed in primary‐care practice, this study provides valuable new information of its epidemiology in this setting. Demographics reported are supportive of previous work and additional novel associations identified, such as the Border terrier, could enhance the index of suspicion for veterinarians.

## INTRODUCTION

Naturally occurring Cushing's syndrome, hereby referred to as Cushing's syndrome, is an endocrine disorder that results from chronic excessive production of glucocorticoids, due to an adrenocorticotropic hormone (ACTH) secreting pituitary mass, a glucocorticoid secreting adrenal tumour, or other rarer aetiologies (Behrend *et al*. 2013, Nelson & Couto 2014). The excessive circulating glucocorticoids, most commonly cortisol, result in the typically recognised clinical signs and are associated with increased risk for several other diseases including diabetes mellitus, hypertension and urolithiasis (Cook *et al*. 1993, Hess *et al*. 2000, Ramsey *et al*. 2005, Fracassi *et al*. 2015, Miceli *et al*. 2017).

Cushing's syndrome is reported to occur in approximately 0.20 to 0.28% of dogs attending primary‐care practice (O'Neill *et al*. 2016, Carotenuto *et al*. 2019). Previously reported estimates have examined the prevalence over varying time periods and across a limited number of practices therefore their wider generalisability is uncertain. A number of risk factors have been reported to be associated with Cushing's syndrome. Sex predisposition is still a contentious risk factor with a number of studies reporting an association with female dogs (Gallelli *et al*. 2010, Hoffman *et al*. 2018, Carotenuto *et al*. 2019). However, these studies are limited by either applying univariable analyses or examining dogs attending referral practices. One study examining primary‐care practice and taking other risk factors into account failed to identify a sex association with Cushing's syndrome (O'Neill *et al*. 2016). Many breeds have been associated with Cushing's syndrome, most notably for the UK primary‐care population, the Bichon frise (O'Neill *et al*. 2016), Yorkshire terrier, Jack Russell terrier (O'Neill *et al*. 2016, Carotenuto *et al*. 2019) and Dachshund (O'Neill *et al*. 2016, Hoffman *et al*. 2018). Other breeds that have been strongly associated with Cushing's syndrome include, but are not limited to, the Schnauzer, Fox terrier, Cavalier King Charles Spaniel, Boxer, Shih‐tzu (Carotenuto *et al*. 2019), Poodle, Irish Setter and Bassett Hound (Hoffman *et al*. 2018). However, some over‐represented breeds reported in the literature are based on very small numbers, or referral dog populations that would likely be a biased sub‐group of the general dog population (Bartlett *et al*. 2010). Also, not all published studies took the underlying breed population into account (Ling *et al*. 1979). Few breeds have been reported with decreased risk of having Cushing's syndrome; the Border Collie and Labrador retriever had decreased odds when compared to crossbreeds in one study (O'Neill *et al*. 2016) and a lower frequency of Cushing's syndrome has been reported in the Border Collie, Rottweiler, Great Dane and Doberman Pinscher in another (Hoffman *et al*. 2018). Further investigation into predisposed and lower risk breeds is warranted to assist primary‐care practitioners in their index of suspicion for Cushing's syndrome and to inform future research, such as genetic studies. Other previously associated risk factors include older age and a greater weight for their breed (O'Neill *et al*. 2016). Before undertaking this study, the authors completed a narrative review of the published literature surrounding the diagnosis of Cushing's syndrome in dogs. Key words in PubMed included Cushing's OR hyperadrenocorticism OR hypercortisolism AND canine OR dog for publications before April 2020. Relevant textbooks were also examined (Feldman & Nelson 2004, Nelson & Couto 2014, Ettinger *et al*. 2017).

The objectives of this study were (1) to estimate the 1‐year period prevalence and incidence for Cushing's syndrome in dogs under UK primary veterinary care and (2) to investigate risk factors for Cushing's syndrome. A further objective was to report data for Cushing's syndrome in dogs under primary veterinary care, which could be used as clinical benchmarks.

Based on the literature examined, the hypotheses of this study were (1) the Bichon frise, Yorkshire terrier, Jack Russell Terrier and Dachshunds would have the highest odds of Cushing's syndrome diagnosis among purebred dogs in the UK primary‐care population and (2) there would be no difference in odds of Cushing's syndrome diagnosis between male and female dogs.

## METHODS

This study used routinely recorded anonymised electronic patient records (EPR) from UK primary‐care veterinary practices collaborating within the VetCompass programme. A retrospective cohort analysis of all dogs under veterinary care at collaborating practices in 2016 was used to report the frequency and to examine the risk factors for dogs diagnosed with Cushing's syndrome. Dogs under veterinary care were defined as having either (1) at least one EPR documented during 2016 or (2) at least one EPR documented during 2015 and 2017. Search terms were applied to the study population to identify candidate dogs with increased probability of Cushing's syndrome: ‘hypera, hyperadr*, hac, cushin*, cushings~1, trilos*, vetor*’. The clinical records of all candidate dogs were manually reviewed to identify dogs eligible for inclusion. A Cushing's syndrome case was included if a pre‐existing (first diagnosed before 2016) or incident (first diagnosed within 2016) diagnosis of Cushing's syndrome was recorded within the EPR between 1 January 2016 and 31 December 2016. Incident cases were additionally required to have a record of a low‐dose dexamethasone suppression test (LDDST) or an ACTH stimulation test being performed within the EPR before diagnosis. Dogs were excluded as a case if the condition was considered iatrogenic or the dog had a record of glucocorticoid administration in the 30 days before first suspicion. All dogs that were not identified by the search terms as candidate cases during the initial screening were included as non‐cases for Cushing's syndrome in the risk factor analysis.

Data were cleaned in Excel (Microsoft Corp.) and uploaded into Stata 15 (Stata, TX, USA) for statistical analysis. Sample size calculations estimated that 187,200 dogs would need to be sampled to provide a prevalence estimate for a disorder expected to occur in 0.2% of the overall UK dog population (estimated at 8 million in 2016), with a 0.02% acceptable margin of absolute error at a 95% confidence level (Asher *et al*. 2011, OpenEpi 2018). Ethical approval was granted by the Royal Veterinary College Ethics and Welfare Committee (SR2018‐1652).

Available demographic data for study dogs included date of birth, sex, neuter status, insurance status, breed, mean lifetime bodyweight above 18 months, veterinary group and veterinary clinic ID. Age (years) was calculated using the date of birth and the date of initial diagnosis for cases, or at the end of the study period (31 December 2016) for non‐cases. Individual purebred and designer breeds were included in the risk factor analysis if there were either (1) greater than or equal to 15 Cushing's syndrome case dogs of that breed or (2) greater than or equal to 5000 non‐case dogs of that breed. All other breeds were grouped as either (1) ‘purebred other’ if they were a recognised breed (VeNom Coding Group 2018) or (2) ‘crossbred’ if they were recorded as a crossbreed or a designer breed‐cross (e.g. Labrador‐poodle or labradoodle). Bodyweight (kg) described the mean from all bodyweight data recorded for dogs aged over 18 months and was split into six categories: less than 10, 10 to less than 20, 20 to less than 30, 30 to less than 40, greater than or equal to 40 and unrecorded. Mean adult bodyweight was calculated for both sexes of each breed with at least 100 dogs in the overall study population to create a variable called ‘bodyweight to breed‐sex mean.’ Dogs were categorised as ‘above’ (over 15% of breed‐sex bodyweight mean), ‘within’ (within 15% of breed‐sex bodyweight mean), ‘below’ (under 15% of breed‐sex bodyweight mean) or ‘unknown’. Neuter and insurance status were recorded at the end of the study period for cases and non‐cases. Individual veterinary clinics attended were assigned identification (ID) numbers. Veterinary groups consisted of clinics that were part of the same group of consolidated clinics and were assigned identification numbers from 1 to 5. Additional descriptive data were extracted by manual revision of the case EPRs, including date of first diagnosis, date of death, referral for Cushing's syndrome, diagnostic testing performed, initial treatment method and recorded underlying aetiology of disease.

Categorical data were presented showing the count and corresponding percentage. Quantitative data were assessed for normality using Shapiro–Wilk testing and graphically; normally distributed data were summarised using the mean [standard deviation (SD)] and non‐normally distributed data using the median [interquartile range (IQR) and range]. A Kaplan–Meier plot described the all‐cause mortality trend of incident cases of Cushing's syndrome from the date of diagnosis. Median survival time (MST) was defined as the number of days for the cumulative percentage of dogs surviving from the date of diagnosis to reach 50% (Dohoo *et al*. 2012). The end date was recorded as the date of death for dogs that were recorded to have died before 30 June 2020. Dogs were censoring at the date of the last clinical record for those that were lost to follow.

To estimate the 1‐year period prevalence of Cushing's syndrome in 2016 within dogs under veterinary care, the proportion of pre‐existing and incident cases was divided by the denominator of all study dogs, overall and within major breeds. Incidence risk was estimated using the incident cases identified in 2016. Confidence interval (CI) estimates were derived from standard errors, based on approximation to the binomial distribution (Kirkwood & Sterne 2010).

Univariable and multivariable binary logistic regression modellings were used to assess associations between risk factors and Cushing's syndrome. Veterinary groups were included in the model as a fixed effect and clinic ID as a random effect to account for clustering within the models. Risk factors with a broad association with the outcome during univariable analysis [likelihood ratio test (LRT)=P<0.2] were considered for multivariable evaluation. Multivariable model‐building used a backwards stepwise manual approach (Dohoo *et al*. 2012). Bodyweight was excluded from multivariable modelling, as it is a defining characteristic of individual breeds, and breed associations with Cushing's syndrome were an a priori variable of interest in this study. The combined variable of sex with neutering status was included in multivariable modelling. Confounding effects were assessed by observing for a greater than 10% change in the ORs following inclusion of an additional risk factor (Katz 2011). Continuous variables were assessed for linearity with the outcome by comparing nested models of the variable, included as either categorical or linear, using a LRT. Where the associations were non‐linear, the relationship for that variable and the diagnosis of Cushing's syndrome were described through graphical assessment; a smoothed line was presented on the logit scale to present the visual association with the binary outcome (Harrell Jr 2015). When modelling a non‐linear variable during risk factor analysis, the term that best modelled the relationship was explored, using quadratic terms, restricted cubic splines and categorisation (Dohoo *et al*. 2012). Akaike Information Criterion (AIC) and Bayesian Information Criterion (BIC) compared the non‐nested models, with smaller IC numbers indicating a better variable model (Dohoo *et al*. 2012, Clayton & Hills 2013). Biologically plausible pairwise interactions in the final model were examined with a LRT (Dohoo *et al*. 2012). Goodness of fit and discrimination of the final model were evaluated by the Hosmer‐Lemeshow test and the area under the receiver operating characteristic (AUROC) curve (Hosmer Jr *et al*. 2013, O'Neill *et al*. 2020). Statistical significance was set at P<0.05.

## RESULTS

The study population included 905,544 dogs under primary veterinary care across 886 VetCompass participating practices in 2016. Search terms identified 12,141 candidate dogs with evidence of consideration of Cushing's syndrome in the EPRs. All candidate dogs were manually examined and 1527 Cushing's syndrome cases were identified; 970 pre‐existing cases to 2016 and 557 incident cases in 2016.

**FIG 1:**
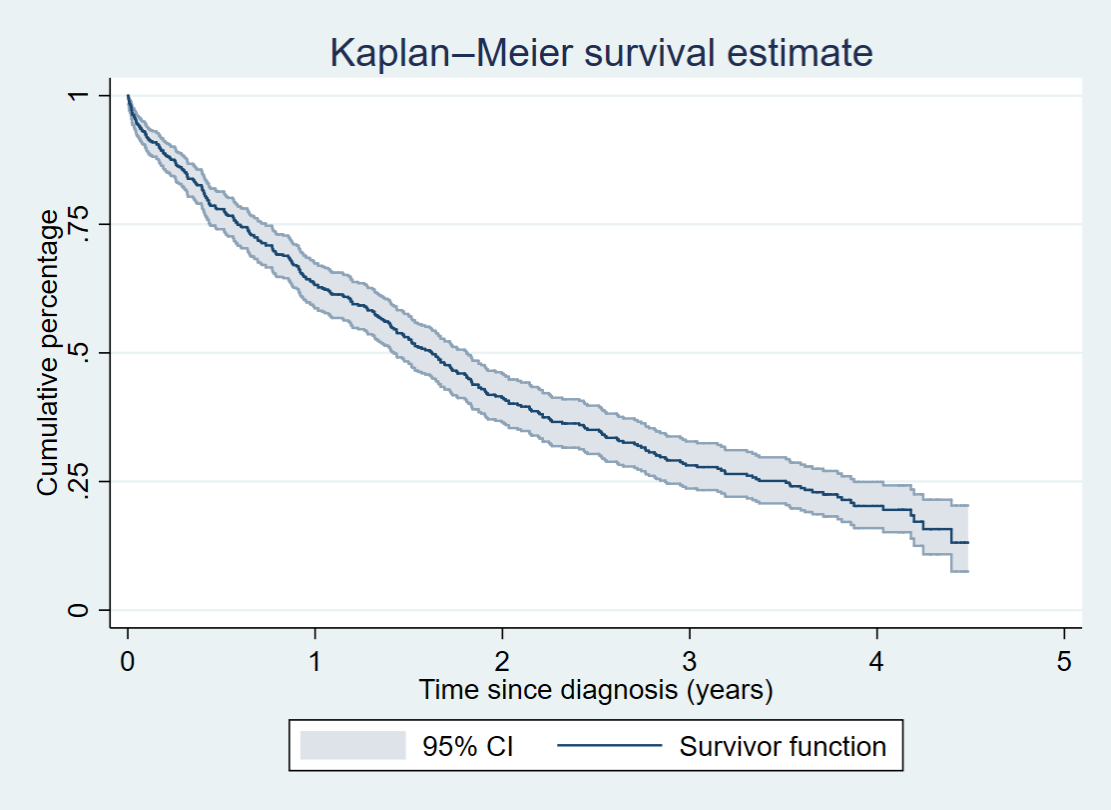
Kaplan–Meier survival curve of all‐cause mortality in dogs diagnosed with Cushing's syndrome at UK primary‐care practices. Survival time represents the time from the date of diagnosis until the date of death (n=557)

Of the 557 incident cases, 51.4% (n=286) were female, 66.1% (368) were neutered and 28.2% (157) were insured. The median age at diagnosis was 10.9 years (IQR 9.0 to 12.7, range 3.9 to 16.1) and median weight was 11.6 kg (IQR 8.8 to 20.0, range 3.6 to 47.7). The most frequently reported breeds were the Jack Russell terrier (n=54/557, 9.7%), Staffordshire Bull terrier (50, 9.0%), Bichon frise (37, 6.7%) and Border terrier (35, 6.3%). Diagnosis was supported using an ACTH stimulation test in 78.6% of incident cases (438/557) and/or 28.9% (161) with a LDDST. Additional tests recorded in the EPRs included the urine cortisol‐to‐creatinine ratio (64, 11.5%), endogenous ACTH assay (2, 0.4%), 17‐OH‐Progesterone (10, 1.8%) and a high dose dexamethasone suppression test (4, 0.7%). Referral was recorded in 14 (2.5%) cases and the underlying aetiology of disease was recorded in 31 (5.6%) cases; 22 (70.1%) had pituitary dependent hypercortisolism (PDH) and 9 (29.9%) had adrenal dependent hypercortisolism (ADH). No other aetiologies for Cushing's syndrome were described within the EPRs. Initial treatment recorded following diagnosis included trilostane (n=508, 91.0%) and adrenalectomy (2, 0.4%). No treatment was instigated in 48 (8.6%) cases. There were 333 (59.8%) cases that died within the study period, 166 (29.8%) were lost to follow‐up and 58 were alive (10.4%) at study end‐point. MST of cases from the date of diagnosis was 594 days (95% CI 519 to 661) (Fig 1). The cumulative proportion of all cases surviving to 1 year was 0.63 (95% CI 0.59 to 0.67) and 0.41 (95% CI 0.37 to 0.46) surviving to 2 years.

### Disease frequency

The estimated 1‐year period prevalence for Cushing's syndrome in dogs in 2016 was 0.17% (95% CI 0.16 to 0.18). The 1‐year incidence risk in 2016 was 0.06% (95% CI 0.05 to 0.07). The breeds with the highest breed prevalence's were the Border terrier (1.02%; 95% CI 0.82 to 1.22), Bichon frise (0.97%; 95% CI 0.80 to 1.13) and Miniature schnauzer (0.42%; 95% CI 0.28 to 0.55) (Table 1).

**Table 1:** Overall estimated prevalence of Cushing's syndrome in dogs under primary veterinary care in the UK, by breed (cases n=1527; population n=905,544)

Category	Prevalence (%)	95% Confidence interval (%)
Border Terrier	1.02	0.82 to 1.22
Bichon frise	0.97	0.80 to 1.13
Miniature schnauzer	0.42	0.28 to 0.55
West Highland White terrier	0.38	0.29 to 0.46
Yorkshire terrier	0.34	0.27 to 0.41
Lhasa apso	0.33	0.23 to 0.44
Jack Russell terrier	0.30	0.25 to 0.34
Boxer	0.26	0.16 to 0.37
Cavalier King Charles Spaniel	0.19	0.12 to 0.25
Staffordshire Bull terrier	0.19	0.15 to 0.23
Beagle	0.16	0.07 to 0.25
Pomeranian	0.16	0.06 to 0.26
English springer spaniel	0.13	0.08 to 0.18
Crossbred	0.12	0.11 to 0.14
Shih‐tzu	0.12	0.08 to 0.15
Cocker spaniel	0.09	0.06 to 0.12
Golden retriever	0.08	0.03 to 0.18
Rottweiler	0.08	0.02 to 0.15
Purebred other	0.07	0.06 to 0.08
Labrador retriever	0.07	0.05 to 0.09
Border Collie	0.05	0.02 to 0.08
Chihuahua	0.02	0.008 to 0.04
French Bulldog	0.01	0.00 to 0.03
German shepherd dog	0.01	0.00 to 0.03
Pug	0.01	0.00 to 0.03
Cockapoo	0.01	0.00 to 0.02

### Risk factor analysis

Univariable analysis identified breed, age at diagnosis, neutering status, sex combined with neutering status, bodyweight to breed‐sex mean and insurance status as strongly associated with Cushing's syndrome (<0.001). Sex alone was weakly associated (P=0.084) and was also taken forward for evaluation in the multivariable analysis (Table 2).

**Table 2:** Descriptive statistics and univariable logistic regression of associated risk factors for Cushing's syndrome in dogs attending UK primary‐care practice in 2016

Variable	Cases	Non‐cases	Odds ratio	95% Confidence Interval	Category P‐value^†^	Variable P‐value^‡^
Sex						
Female	286 (51.4)	425,630 (47.9)	‐			0.084
Male	271 (48.6)	463,568 (52.1)	0.86	0.73 to 1.02	0.084	
Neuter status						
Entire	189 (33.9)	489,512 (55.1)	‐			<0.001
Neutered	368 (66.1)	399,688 (44.9)	2.21	1.85 to 2.65	<0.001	
Insurance status						<0.001
Insured	157 (28.2)	114,306 (12.8)	2.27	1.84 to 2.81	<0.001	
Uninsured	400 (71.8)	779,096 (87.2)	‐			
Breed						
Crossbred	117 (21.1)	225,304 (25.3)	‐			<0.001
Border terrier	35 (6.3)	9317 (1.1)	7.11	4.86 to 10.39	<0.001	
Bichon frise	37 (6.7)	12,762 (1.4)	5.56	3.83 to 8.06	<0.001	
Lhasa apso	16 (2.9)	12,365 (1.4)	2.71	1.60 to 4.59	<0.001	
Miniature schnauzer	12 (2.2)	8133 (0.9)	2.71	1.49 to 4.92	0.001	
West Highland White terrier	24 (4.3)	18,136 (2.0)	2.51	1.62 to 3.91	<0.001	
Yorkshire terrier	32 (5.8)	27,413 (3.1)	2.23	1.51 to 3.30	<0.001	
Jack Russell terrier	54 (9.7)	47,641 (5.4)	2.17	1.57 to 3.00	<0.001	
Staffordshire Bull terrier	50 (9.0)	52,285 (5.9)	1.85	1.33 to 2.58	<0.001	
Boxer	8 (1.4)	9203 (1.0)	1.66	0.81 to 3.40	0.167	
Cavalier King Charles Spaniel	14 (2.5)	16,967 (1.9)	1.55	0.89 to 2.70	0.124	
Rottweiler	5 (0.9)	7233 (0.8)	1.34	0.55 to 3.27	0.526	
Purebred other	94 (16.9)	114,406 (16.2)	1.24	0.94 to 1.62	0.129	
Beagle	5 (0.9)	7949 (0.9)	1.22	0.50 to 2.98	0.669	
English springer spaniel	8 (1.4)	19,978 (2.3)	0.77	0.38 to 1.58	0.477	
Shih‐tzu	11 (2.0)	32,603 (3.7)	0.68	0.37 to 1.26	0.22	
Cocker spaniel	8 (1.4)	31,804 (3.6)	0.47	0.23 to 0.97	0.041	
Border Collie	5 (0.9)	22,178 (2.5)	0.44	0.18 to 1.08	0.075	
Labrador retriever	13 (2.3)	59,279 (6.7)	0.41	0.23 to 0.73	0.002	
Golden retriever	2 (0.4)	9647 (1.1)	0.37	0.09 to 1.49	0.16	
Pomeranian	1 (0.2)	6147 (0.7)	0.31	0.04 to 2.21	0.241	
French Bulldog	1 (0.2)	16,337 (1.8)	0.12	0.02 to 0.87	0.036	
Pug	1 (0.2)	16,153 (1.8)	0.12	0.02 to 0.88	0.037	
Cockapoo	1 (0.2)	18,234 (2.1)	0.11	0.01 to 0.76	0.025	
Chihuahua	1 (0.2)	36,671 (4.1)	0.06	0.01 to 0.40	0.004	
German shepherd dog	0 (0.0)	21,238 (2.4)	~	~	~	
Weight						
<10	178 (32.0)	211,351 (23.7)	‐			<0.001
10 to <20	188 (33.8)	162,797 (18.2)	1.35	1.10 to 1.66	0.004	
20 to <30	76 (13.6)	116,972 (13.1)	0.76	0.58 to 0.99	0.042	
30 to <40	31 (5.6)	67,514 (7.6)	0.53	0.36 to 0.78	0.001	
≥40	14 9 (2.5)	25,786 (2.9)	0.63	0.36 to 1.08	0.093	
Missing	70 (12.6)	308,982 (34.6)	0.15	0.10 to 0.21	<0.001	
Weight to breed‐sex mean						
Below	104 (18.7)	171,305 (19.2)	0.80	0.63 to 1.01	0.062	<0.001
Within	203 (36.5)	265,045 (29.7)	‐	‐		
Above	179 (32.1)	145,962 (16.3)	1.60	1.31 to 1.96	<0.001	
Missing	71 (12.8)	311,090 (34.8)	0.17	0.11 to 0.24	<0.001	
Sex‐neuter						
Male entire	105 (18.9)	257,400 (29.0)	‐			<0.001
Male neutered	166 (29.8)	206,168 (23.2)	1.84	1.44 to 2.35	<0.001	
Female entire	84 (15.1)	232,110 (26.1)	0.89	0.67 to 1.19	0.444	
Female neutered	202 (36.3)	193,520 (21.7)	2.38	1.88 to 3.02	<0.001	
Age median (IQR, years)	10.9 (9.0 to 12.7)	4.4 (1.8 to 7.9)				<0.001
Linear term			4.18	3.44 to 5.07	<0.001	
Quadratic term			0.95	0.94 to 0.95	<0.001	

Veterinary clinic ID was included as a random effect (cases n=557; non‐cases n=893,403)

^†^ Wald P‐value

^‡^ Likelihood ratio test P‐value

The final breed‐focussed multivariable model included four variables: breed, age at diagnosis, bodyweight to breed‐sex mean and insurance status. Seven breeds were associated with increased odds of Cushing's syndrome; the Bichon frise (OR=6.17, 95% CI 4.22 to 9.00), Border terrier (5.40, 95% CI 3.66 to 7.97), Miniature schnauzer (3.05, 95% CI 1.67 to 5.57), Lhasa apso (2.52, 95% CI 1.49 to 4.28), Yorkshire terrier (1.82, 95% CI 1.23 to 2.70), Staffordshire Bull terrier (1.52, 95% CI 1.08 to 2.13) and Jack Russell Terrier (1.50, 95% CI 1.07 to 2.08). Four breeds were at decreased odds of Cushing's syndrome: the Golden retriever (0.24, 95% CI 0.06 to 0.98), Labrador retriever (0.3, 95% CI 0.17 to 0.54), Border Collie (0.32, 95% CI 0.13 to 0.78) and Cocker spaniel (0.44, 95% CI 0.21 to 0.90). Dogs with a bodyweight higher than their breed‐sex mean had 1.44 times the odds of Cushing's syndrome than those within their breed mean (95% CI 1.17 to 1.78; P<0.001). Dogs that were insured were associated with greater odds of Cushing's syndrome compared to uninsured dogs (OR=2.46, 95% CI 1.98 to 3.04; P<0.001) (Table 3).

**Table 3:** Multivariable logistic regression analysis to assess the risk factors for Cushing's syndrome in dogs under primary veterinary care in the UK in 2016

	Odds ratio	95% Confidence Interval	Category P‐value^†^	Variable P‐value^‡^
Breed				<0.001
Crossbred	‐	‐	‐	
Bichon frise	6.17	4.22 to 9.00	<0.001	
Border terrier	5.40	3.66 to 7.97	<0.001	
Miniature schnauzer	3.05	1.67 to 5.57	<0.001	
Lhasa apso	2.52	1.49 to 4.28	0.001	
Yorkshire terrier	1.82	1.23 to 2.70	0.003	
Beagle	1.77	0.72 to 4.36	0.214	
Rottweiler	1.53	0.62 to 3.76	0.360	
Staffordshire Bull terrier	1.52	1.08 to 2.13	0.017	
Jack Russell terrier	1.50	1.07 to 2.08	0.016	
Boxer	1.35	0.66 to 2.79	0.414	
West Highland White terrier	1.32	0.84 to 2.06	0.225	
French Bulldog	1.30	0.21 to 10.87	0.686	
Purebred other	1.26	0.95 to 1.66	0.112	
Cavalier King Charles Spaniel	1.24	0.71 to 2.17	0.450	
Cockapoo	0.93	0.13 to 6.69	0.940	
Shih‐tzu	0.89	0.48 to 1.66	0.713	
Pomeranian	0.63	0.09 to 4.54	0.649	
English springer spaniel	0.55	0.27 to 1.14	0.106	
Cocker spaniel	0.44	0.21 to 0.90	0.024	
Pug	0.37	0.21 to 10.87	0.318	
Border Collie	0.32	0.13 to 0.78	0.012	
Labrador	0.30	0.17 to 0.54	<0.001	
Golden retriever	0.24	0.06 to 0.98	0.048	
Chihuahua	0.15	0.02 to 1.10	0.062	
German shepherd dog	^§^	^§^	^§^	
Weight to breed‐sex mean				<0.001
Under	0.98	0.76 to 1.25	0.850	
Within	‐		‐	
Over	1.44	1.17 to 1.78	0.001	
Missing	0.40	0.27 to 0.58	<0.001	
Insurance status				<0.001
Insured	2.46	1.98 to 3.04	<0.001	
Uninsured	‐	‐	‐	
Age				<0.001
Linear term	4.10	3.35 to 5.01	<0.001	
Quadratic term	0.95	0.94 to 0.96	<0.001	

Veterinary group included as a fixed effect and veterinary clinic as a random effect (cases n=557; non‐cases n=853,704)

^†^ Wald P‐value

^‡^ Likelihood ratio test P‐value

^§^ No data

**FIG 2:**
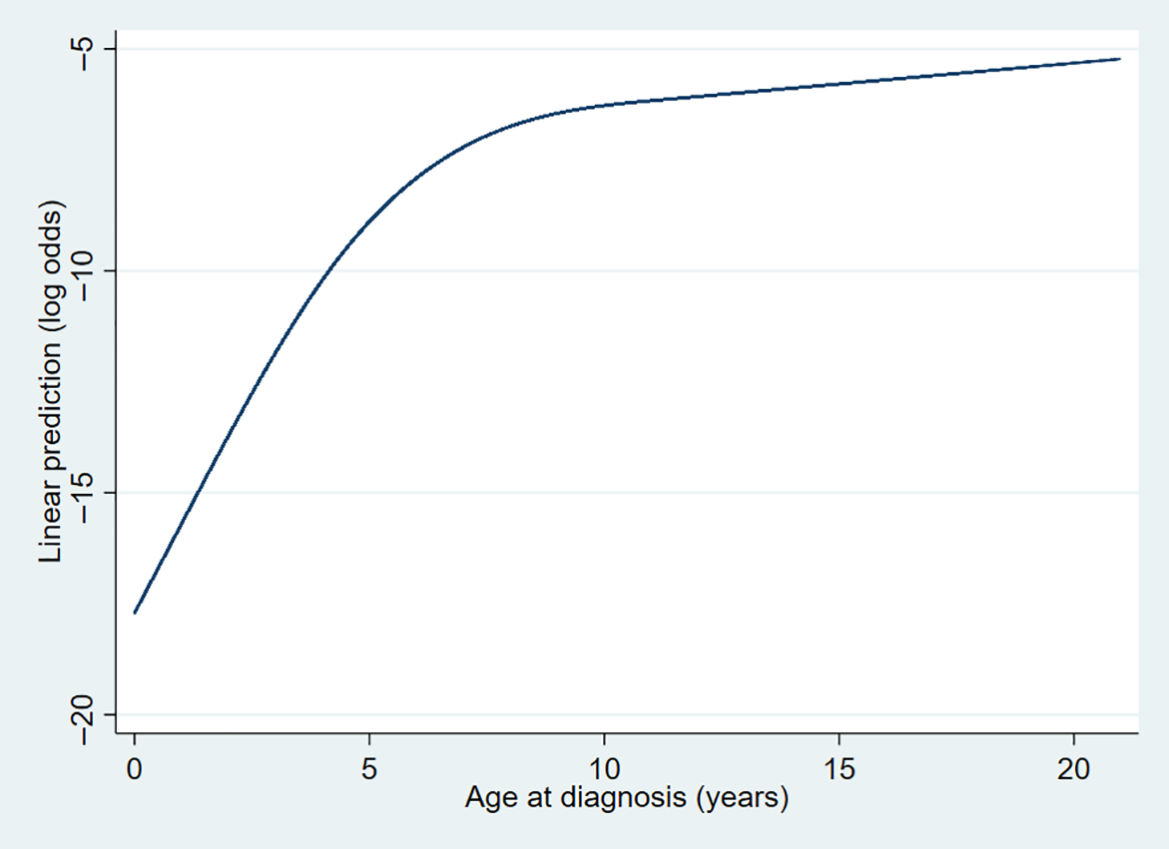
Smoothed line on the logit scale presenting the non‐linear association of age at diagnosis in dogs with Cushing's syndrome under primary veterinary care in the UK (n=557)

The non‐linear association of age with Cushing's syndrome was explored through visual interpretation of a smoothed line on the logit scale (Fig 2). Overall, the odds of Cushing's syndrome increased with increasing age. The relationship demonstrated a rapid increase in the odds of developing Cushing's syndrome up until the age to around 7 years of age. The increasing odds of Cushing's syndrome then only increased very gradually from 7 years onwards. To account for the confounding effect of age in logistic regression modelling, age was included as a quadratic term as this best described the non‐linear relationship.

Veterinary clinic ID was statistically significant and was included as a random effect indicating significant variation at the clinical level (LRT of rho P<0.001, rho=0.08). The final model demonstrated acceptable model fit (Hosmer lemeshow p=0.265) and model discrimination (AUROC=0.904; 95% CI 0.896 to 0.912). Sex‐neuter was not retained in the multivariable model as the univariable effects were confounded fully by age and insurance status (P=0.302). No pair‐wise interactions were identified.

## DISCUSSION

This is the largest study to date to estimate the prevalence, incidence and risk factors for Cushing's syndrome in dogs attending primary‐care practice. One‐year period prevalence for Cushing's syndrome in 2016 within UK primary‐care practice was estimated at 0.17%. Previous estimates within primary‐care populations of 0.20% (95% CI 0.13 to 0.27) and 0.28% (95% CI 0.25 to 0.31) included dogs from over 2‐ and 6‐year time periods respectively (O'Neill *et al*. 2016, Carotenuto *et al*. 2019). The prevalence in the current study is slightly lower than those previously reported, however the CIs across the estimates within primary‐care populations overlap and could be explained by the shorter time period observed in the current study. Studying prevalence across a longer time period provides dogs with a longer amount of time to become a case and therefore are likely to report higher prevalence than studies with a shorter time period (Ng *et al*. 2013, Ward 2013). Other published studies also utilising data from VetCompass have reported a 1‐year period prevalence in 2016 therefore allowing direct comparison to other disorders (Heeley *et al*. 2020, Schofield *et al*. 2021). Prevalence estimates within referral level practice suggest a higher Cushing's syndrome prevalence of 1.2 to 1.5% within a referral centre caseload (Bellumori *et al*. 2013, Carotenuto *et al*. 2019).

The annual incidence risk was estimated at 0.06% (95% CI 0.05 to 0.07). Incidence risk has been estimated for Cushing's syndrome in two previous studies: 96 of 100,000 dogs/year (equivalent to 0.096%) in a UK study of insured dogs (Dobson *et al*. 2002) and one to two cases of 1000 dogs/year (0.1 to 0.2%) of pituitary dependent disease in USA veterinary hospitals (Willeberg & Priester 1982). Drawing direct comparisons across the studies is difficult due to the different source populations used; however, the present study is likely the most accurate current estimation of Cushing's syndrome incidence within UK primary‐care practice. Based on the frequency estimates within the literature, it is clear that Cushing's syndrome is an infrequently diagnosed disease within primary‐care veterinary practice and appears to be encountered less frequently for veterinarians in primary‐care practice than for those within referral practices. Finally, prevalence may vary with geography, given that in human and veterinary medicine pituitary tumour aetiopathogenesis has been associated with exposure to environmental factors (Dirtu *et al*. 2013, Cannavo *et al*. 2016).

Data that could be useful as clinical benchmarks were presented on the clinical approaches and survival characteristics of Cushing's syndrome in primary‐care practice. The ACTH stimulation test was performed in 78.6% of incident cases and was used more than the LDDST in the present study. The ACTH stimulation test has previously been reported to be used in greater than 90% of cases within primary‐care practice in the UK (O'Neill *et al*. 2016, Schofield *et al*. 2020). Other diagnostic tests for Cushing's syndrome were also used less frequently in the current study to previously reported benchmarking parameters (O'Neill *et al*. 2016). Previous studies examined earlier time periods (before 2013) and a smaller sample of UK veterinary practices, which could explain these differences. The commonly used diagnostic tests for Cushing's syndrome are not highly specific resulting in false positive results. The ACTH stimulation test has a likely specificity of 59 to 61% in dogs suspected of Cushing's syndrome (Monroe *et al*. 2012, Nivy *et al*. 2018), indicating a proportion of false positives in this study are likely. Equally, the ACTH stimulation test lacks sensitivity, estimates range from 80 to 88% for PDH cases to 57 to 63% for ADH cases (Peterson *et al*. 1982, Feldman 1983, Reusch & Feldman 1991, Kaplan *et al*. 1995, Monroe *et al*. 2012). Therefore, cases could be falsely excluded when using the ACTH stimulation. Specific aspects about the ACTH stimulation test protocols used by veterinarians in the current study were not known. The type of synthetic ACTH administered (Hill *et al*. 2004), the cortisol assay used (Behrend *et al*. 2013, Bennaim *et al*. 2019) and the sample collection method (Behrend *et al*. 1998, Schechter *et al*. 2020) can influence ACTH stimulation test cortisol concentrations. Further research into the ACTH stimulation test protocols used by primary‐care veterinarians would be beneficial to be able to identify and address any factors that could improve interpretation of this preferred primary‐care test. No diagnostic tests currently available for the diagnosis of Cushing's syndrome are highly accurate, however, alternative tests such as the LDDST and dynamic tests based on measuring UCCR are more sensitive than the ACTH stimulation test (Feldman 1983, Rijnberk & Mol 1988, Galac *et al*. 1997, Bennaim *et al*. 2018). Had the use of these alternative tests been greater in the current study population, prevalence estimates could have been higher. Generally, novel diagnostic methods are warranted to improve the diagnostic accuracy of Cushing's syndrome.

Referral was recorded in 14 (2.5%) cases, indicating that Cushing's syndrome is a disease that is often diagnosed and managed completely within the primary‐care setting. This highlights the importance of examining primary‐care populations for this disease. The referral dog population are likely to consist of more complicated cases; therefore, assessment of diagnostic and monitoring test accuracy, and drug effectiveness for Cushing's syndrome using cases from within referral populations may not be applicable to the wider dog population. Further studies utilising cases of Cushing's syndrome from within primary‐care populations are warranted to increase the generalisability of the evidence surrounding this disease. Differentiation into the underlying aetiology of Cushing's syndrome was rarely recorded, in only 5.6% of cases. This is lower than the 21.1% previously reported in a primary‐care population in the UK (Schofield *et al*. 2019a). Differences in the veterinary practices studied could describe some of this disparity, with variations in owner, veterinarian or practice demographics having potential effects. Cases that were differentiated were predominantly based on interpretation of ultrasonographic imaging of the adrenals or through interpretation of the LDDST. The LDDST is not a reliable method of identifying ADH (Feldman *et al*. 1996, Bennaim *et al*. 2018, van Bokhorst *et al*. 2019) and adrenal measurement via ultrasonography is considered difficult (Grooters *et al*. 1996); therefore, there is a possibility of incorrect reporting of the underlying aetiology under primary veterinary care. Reasons for infrequent differentiation could be explained by 91% of dogs receiving medication with trilostane following diagnosis, regardless of the underlying aetiology and surgical treatment rarely undertaken. The long‐term management of cases in this study was not followed up over time; therefore, the number of dogs receiving surgical treatment at some point within their clinical records could have been slightly higher.

The MST in the current study of 594 days (95% CI 519 to 661) is in line with previous estimates within the literature in dogs treated with trilostane (Barker *et al*. 2005, Clemente *et al*. 2007, Arenas *et al*. 2014, Fracassi *et al*. 2015, Nagata *et al*. 2017, Schofield *et al*. 2019a). This shows reasonable survival in dogs with Cushing's syndrome attending primary‐care practice and the 1 and 2 year survival proportions indicated the over 60 and 40% survived to these time points respectively. This reflects a considerable amount of time for dogs to be living with Cushing's syndrome and highlights the need to reduce the impact of the associated clinical signs and improve their quality‐of‐life during this time (Schofield *et al*. 2019b). This is also useful information to provide to owners, to support them following their dog's diagnosis. Survival estimates for dogs managed with trilostane from referral populations are slightly more optimistic; however, differences may exist in the dog populations, such as mean age, breed differences and other comorbidities that could account for the differences observed. Survival estimates in the current study include 48 (8.6%) dogs that did not receive treatment and could have shortened the median survival (Nagata *et al*. 2017, Schofield *et al*. 2019a). Additionally, survival estimates for ADH are inferior to PDH; therefore, differing proportions of ADH and PDH in these populations could account for encountered prevalence differences (Barker *et al*. 2005, Clemente *et al*. 2007, Helm *et al*. 2011, Arenas *et al*. 2014, Fracassi *et al*. 2015, Nagata *et al*. 2017).

Breed, age at diagnosis, bodyweight to breed‐sex mean and insurance status were associated with the diagnosis of Cushing's syndrome in dogs attending primary‐care practice. A previous study reported the risk factors for combined caseload of incident and pre‐existing cases, making interpretation difficult due to the incurred selection bias from including the pre‐existing cases (O'Neill *et al*. 2016). The identified risk factors and effect measures reported by this prior study could describe the diagnosis and consequent survival for Cushing's syndrome, rather than solely for diagnosis.

Among the breeds, the bichon frise had the highest odds of Cushing's syndrome (OR=6.17), accounting for about one in every 12 incident cases of Cushing's syndrome within primary‐care practice. Six other breeds had increased odds of Cushing's syndrome compared to crossbred dogs, with novel associations of increased odds of Cushing's syndrome in the Border terrier, Lhasa apso and Staffordshire Bull terrier. Based on the literature examined, this is the first time these breeds have been reported. The Border terrier had the highest breed prevalence of Cushing's syndrome and second highest odds in the current study (OR 5.40, 95% CI 3.66 to 7.97). Due to the strong association identified here, it is interesting the Border terrier has not previously been associated with the diagnosis of Cushing's syndrome. The popularity of Border terriers has decreased in England between 2005 and 2014; therefore, this could suggest a more concentrated breeding population in this breed and may be important to monitor in the future (O'Neill *et al*. 2017).

Breeds with reduced odds of Cushing's syndrome compared to crossbred dogs included the Golden retriever, Labrador retriever, Border Collie and Cocker spaniel. The Border Collie consistently appears to be at reduced risk of Cushing's syndrome (O'Neill *et al*. 2016, Hoffman *et al*. 2018) and the Labrador retriever has also previously been identified (O'Neill *et al*. 2016). The Golden retriever and the Cocker spaniel, however, have not previously been reported at reduced odds. Interestingly, of the 21,238 German shepherd dog (GSD) in the underlying population, none were included as incident cases in this analysis. Prior GSD OR estimates vary in the literature from 0.30 (95% CI 0.10 to 1.00) (O'Neill *et al*. 2016) to 1.43 (95% CI 0.34 to 6.00) (Carotenuto *et al*. 2019). Variations in GSD associations could possibly reflect genetic differences within the breed across different countries or could suggest that owners of GSDs within the current study were less likely to pursue a diagnosis of Cushing's syndrome than in other populations. Further research to investigate barriers to assigning a diagnosis of Cushing's syndrome could be beneficial and could highlight possible confounding factors that have not been accounted for in the present study. Additionally the low numbers of Cockerpoos, French Bulldogs and Pugs with Cushing's syndrome should be noted, even with large numbers of dogs included in the underlying population. These three breeds were not significant in the final model after accounting for age due to the rising popularity of these breeds and young populations. For understanding which breeds are predisposed to, and protected from, Cushing's syndrome is important; for veterinarians in primary‐care practice to consider when selecting dogs for specific diagnostic testing for Cushing's syndrome and to guide future research, such as genetic analysis of Cushing's syndrome in dogs (Denyer *et al*. 2020).

Increasing age was associated with increasing odds of Cushing's syndrome in dogs and median age at diagnosis of 10.9 years was in line with other studies (Helm *et al*. 2011, O'Neill *et al*. 2016, Nagata *et al*. 2017, Carotenuto *et al*. 2019). The association with age was not linear, therefore was modelled as a non‐linear term to best control for confounding effects of age on the other risk factors included in the model (Austin & Brunner 2004, Altman & Royston 2006, Royston *et al*. 2006). The quadratic term best described the non‐linear relationship (Dohoo *et al*. 2012). A visual interpretation for age association with Cushing's syndrome was included to aid interpretation (Harrell Jr 2015). The relationship demonstrated the highest odds of Cushing's syndrome from 7 years onwards and can aid veterinarians when considering Cushing's syndrome cases by helping to raise the index of suspicion in older dogs.

Dogs with a bodyweight higher than their breed‐sex mean had 1.44 times the odds of Cushing's syndrome that those within their breed mean. A similar association was reported previously in dogs (O'Neill *et al*. 2016) and the human medical literature, suggesting an association between obesity and increased risk of Cushing's syndrome (Tiryakioglu *et al*. 2010). The mean bodyweight in the current study was calculated across each dog's lifetime from 18 months of age until 31 December 2016. Dogs with a weight greater than the breed‐sex average might suggest obesity or larger examples of the breed as a possible risk factor for Cushing's syndrome. The causal inference is difficult here due to the temporality of observation as a clinical feature of Cushing's syndrome can be weight changes. A gain in weight can result from glucocorticoid impact on central hypothalamic appetite centres resulting in increased appetite and therefore increased calorie consumption (Tataranni *et al*. 1996, McGavin & Zachary 2006). However, excess glucocorticoids also have a catabolic effect on skeletal muscle leading to muscle wastage and weight loss (McGavin & Zachary 2006). Therefore further research is required to understand the temporal sequence of this association and to understand the impact of Cushing's syndrome on bodyweight measurements.

Insured dogs were associated with 2.46 times greater odds of Cushing's syndrome compared to uninsured dogs. The reason for this association could be that the financial benefit of pet insurance might make reaching a diagnosis more likely (Egenvall *et al*. 2009). The higher odds in the insured population also suggest that there may be many uninsured dogs with Cushing's syndrome not identified in the current study. Veterinary group attended was forced into the model as a fixed effect to adjust for variation between groups, but was only weakly associated with Cushing's syndrome in the final model. After taking breed and age into account, the larger effects in the univariable analysis were diluted suggesting variations in pet demographics across the different veterinary groups. Combined sex‐neuter status variable was not associated with Cushing's syndrome in the final model (P=0.302). Studies vary in demonstrating a sex predisposition to Cushing's syndrome. No sex predisposition in dogs was indicated in a study of UK dogs attending primary‐care practice in a multivariable model (O'Neill *et al*. 2016). Female dogs have been associated with Cushing's syndrome in other studies but these all were examining univariable associations; therefore, findings may have reflected confounding rather than true effects (Gallelli *et al*. 2010, Hoffman *et al*. 2018, Carotenuto *et al*. 2019). The current study adds further evidence to there being no major sex association with Cushing's syndrome in dogs. Other factors associated with Cushing's syndrome have been reported but were not assessed in the present study, including concurrent diseases such as diabetes mellitus, biliary mucocoeles and systemic hypertension (Kim *et al*. 2017, Miceli *et al*. 2017, Hoffman *et al*. 2018, García San José *et al*. 2020). The direct causal relationships between Cushing's syndrome and these associated diseases are not well understood. Additional studies utilising primary‐care data could provide further insight into these associations and gain additional understanding of the risk factors for Cushing's syndrome diagnosis.

There were some limitations to this study. The data were retrospective and not primarily recorded for research purposes. There was some missing data in this study and misclassification of Cushing's syndrome was possible; some dogs with Cushing's syndrome might have been included within the denominator population, as a diagnosis could have been missed in primary‐care practice or a diagnosis not sought by an owner. The association of a Cushing's syndrome diagnosis with insurance status highlights this. Dogs with a recorded suspicion of Cushing's in the EPRs were included in the candidate search terms and were excluded from analysis, to minimise this bias. Additionally, there is a risk of dogs being incorrectly diagnosed with Cushing's syndrome in the EPRs and included as a case in this study. For both the ACTH stimulation test and the LDDST, specificity estimates not highly accurate indicating that some falsely diagnosed cases of Cushing's syndrome were likely included in the present study (Van Liew *et al*. 1997, Monroe *et al*. 2012, Bennaim *et al*. 2018, Nivy *et al*. 2018). As misclassification was likely to have occurred for both cases and non‐cases, it was assumed that any incurring bias would likely have resulted in diluting the effect estimates (Dohoo *et al*. 2012).

In conclusion, this study examined a large sample of dogs with Cushing's syndrome diagnosed in 2016 under primary‐care veterinary practices from across the UK. Findings can help veterinarians during diagnosis of Cushing's syndrome by raising awareness of key breed and age associations. Identification of protected breeds offers new options for research into genetic mechanisms. Additionally, owners can be reassured by the promising median survival following diagnosis of Cushing's syndrome, of almost 2 years.

### Funding

IS is supported at the RVC by an award from Dechra Ltd.

### Conflict of interest

IS is supported at the RVC by an award from Dechra Ltd. SN has undertaken consultancy work for Dechra Ltd. The remaining authors have no conflicts of interest to declare.
